# Implementing low-cost 3D-printed brain coloring activities in neuroanatomy teaching for medical students in Singapore: a cross-sectional study

**DOI:** 10.3352/jeehp.2026.23.5

**Published:** 2026-03-18

**Authors:** Jason Wen Yau Lee, Fernando Bello, Jai Prashanth Rao

**Affiliations:** 1 Technology Enhanced Learning and Innovation Department, Duke-NUS Medical School, National University of Singapore, Singapore; 2 National Neuroscience Institute, Singapore; The Catholic University of Korea, Korea

**Keywords:** Neuroanatomy, Three-dimensional printing, Medical education, Educational technology, Singapore

## Abstract

**Purpose:**

Three-dimensional (3D)–printed models have been increasingly used in medical education, but most studies have focused on satisfaction or outcomes following isolated learning activities. This study aimed to explore students’ perceptions of learning, engagement, usability, and learning strategies after completing a series of neuroanatomy-related coloring activities using a low-cost 3D-printed model.

**Methods:**

This cross-sectional study involved Year 1 medical students at Duke-NUS Medical School. Students participated in 3 structured coloring activities using a modular 3D-printed brain model during a neuroanatomy session. An anonymous survey was administered 1 week after the third activity to assess students’ perceived learning value, engagement (behavioral, cognitive, emotional, and agentic), usability, and learning strategies using Likert-scale items and open-ended questions.

**Results:**

A total of 48 students completed the survey, and the instrument showed acceptable to high internal consistency. Students reported high perceived learning value, positive engagement across multiple domains during the coloring activity, and high usability of the model. Participation in the learning activities was associated with significantly higher behavioral and agentic engagement, perceived learning value, and greater use of learning strategies than non-participation. Overall, active manipulation and hands-on exploration were perceived as beneficial for learning.

**Conclusion:**

Low-cost 3D-printed brain models may serve as valuable learning tools to complement existing anatomy teaching approaches when paired with well-designed learning activities. Students reported positive learning experiences and high engagement during the activities. These findings highlight the importance of sound pedagogical design and curriculum integration to maximize learning.

## Graphical abstract

[Fig f2-jeehp-23-05]

## Introduction

### Background

The use of 3-dimensional (3D) printing in teaching and learning has attracted increasing interest over the past decade, particularly in medical education [1]. This interest has been driven not only by the pedagogical potential of 3D-printed models but also by the growing availability of low-cost 3D printers, such as fused deposition modeling (FDM) printers. These developments have enabled educators to create customized, durable 3D-printed models that better meet teaching and learning needs aligned with the curriculum [2].

A major challenge in anatomy education is helping students visualize spatial relationships using 2-dimensional diagrams [3]. Understanding these spatial connections enhances retention by allowing learners to visually and physically connect anatomical parts. Examining an object from different angles, including rotating and manipulating it, helps students develop a clear mental model rather than relying solely on flat images [4]. This kind of 3D exploration may decrease cognitive load and strengthen spatial reasoning in ways that are not apparent with traditional illustrations. Research indicates that using 3D-printed models can lead to improved learning outcomes [5], greater enjoyment [6], and higher satisfaction than conventional methods [7].

Few studies have examined how students engage with 3D-printed models, as most research has focused on isolated instructional activities [8]. This creates an important gap, because engagement is known to promote deep learning, especially in complex subjects such as neuroanatomy. Engagement is often conceptualized as a multidimensional construct encompassing behavioral, cognitive, emotional [9], and agentic [10] dimensions. Behavioral engagement involves the actions students take to remain focused on a task. Cognitive engagement refers to the mental effort students invest in linking new information to prior knowledge. Emotional engagement refers to students’ affective responses to the learning experience. Agentic engagement is a relatively recent dimension that involves students taking initiative in their own learning. Understanding how students engage may provide educators with deeper insight into the pedagogical value of using 3D-printed models for learning.

### Objectives

This study aimed to investigate the educational value of coloring a low-cost FDM-printed 3D brain model for students learning neuroanatomy. It focused on students’ perceived learning value, engagement, usability, and learning strategies. The study also examined differences in perceptions and engagement between students who completed the activity and those who did not.

## Methods

### Ethics statement

This study was approved by the Duke-NUS Departmental Ethics Review Committee (No. 000158).

### Study design

The authors conducted a cross-sectional study to explore students’ experiences with a low-cost 3D-printed brain model incorporated into the neuroanatomy section of the course. Quantitative and qualitative data were collected through an anonymous survey completed 1 week after 3 lessons in November 2025. Results are presented in accordance with the Strengthening the Reporting of Observational Studies in Epidemiology (STROBE) guidelines [11].

### Setting

**Fig. 1.**
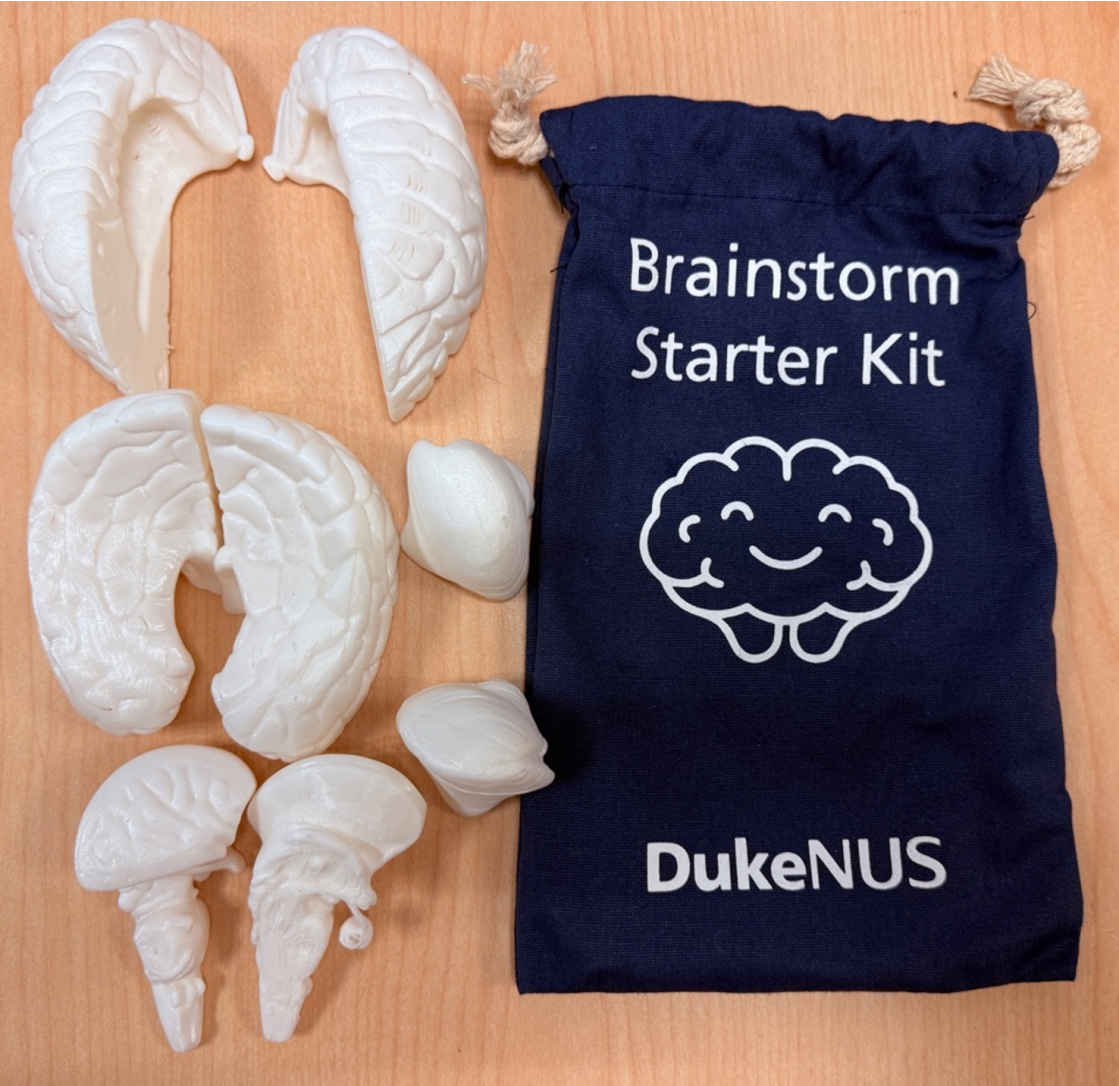
8-part 3-dimensional–printed brain model distributed to students.

This study was conducted at Duke-NUS Medical School in Singapore. Students were enrolled in the Doctor of Medicine (MD) program. All 78 students in the Year 1 cohort received an FDM-printed 8-part brain model (Fig. 1), which cost approximately 8 Singapore dollars (about US$6.50) and required 11 hours to print. As part of the course, students participated in 3 structured coloring sessions using the 3D-printed brain model, each conducted during the final 10 minutes of 3 neuroanatomy lessons. Details of the instructions are provided in Supplement 1.

The activities were not assessed, and students were given specific instructions to color the anatomical structures based on content learned earlier in the lesson to reinforce their understanding of neuroanatomy and its spatial relationships. Students were invited to post images of their completed activities in Microsoft Teams to facilitate sharing and reflection.

### Participants

Participants were recruited from Year 1, Semester 1 of the MD program during the “Foundations of Patient Care 1” course, a pre-clerkship course covering basic sciences, including anatomy, physiology, biochemistry, pharmacology, and pathology.

### Variables

The survey instrument gathered students’ perceptions of learning value, engagement (behavioral, cognitive, emotional, and agentic), usability, and learning strategies related to use of the 3D-printed model. Details of the survey questionnaire can be found in Supplement 2.

### Data sources/measurement

The survey consisted of 20 Likert-scale items scored on a 5-point scale (1=strongly disagree, 5=strongly agree). The items were developed by the first author and grouped into theory-informed dimensions derived from medical education [12] and educational psychology [9]. The items were reviewed for content relevance and clarity by the co-authors, who have expertise in medical education, simulation, and anatomy teaching. Because this instrument was developed for this specific study context and has not been previously validated, its dimensional structure should be considered theory-informed rather than empirically validated. Perceived learning value was measured using 4 items; behavioral, cognitive, emotional, and agentic engagement using 2 items each; and usability and learning strategies using 4 items each. In addition, 2 open-ended questions collected qualitative feedback on suggestions and future enhancements.

### Bias

To minimize bias, no identifiable information, such as name, age, or gender, was collected in the survey. We acknowledge the possibility of response bias because participation in the survey was voluntary.

### Statistical methods

Statistical analysis was performed using IBM SPSS Statistics ver. 29.0 for Mac (IBM Corp.). Survey items were organized according to theory-informed dimensions, and dimension-level scores were calculated by averaging item responses. Internal consistency was assessed using Cronbach’s alpha to evaluate item coherence within the theory-informed groupings. Because this was an exploratory study, no factor analysis was performed. The instrument has not been previously validated, and the dimensional structure should therefore be interpreted as theory-informed rather than empirically confirmed.

An exploratory comparison was conducted to examine differences between students who completed the activity and those who did not, based on self-reports. Because of unequal group sizes, non-normal distributions in several dimensions (Shapiro-Wilk P<0.05), and the ordinal nature of the Likert-derived data, the Mann-Whitney U test was used to compare dimension-level scores between groups. Cohen’s d was calculated as the mean difference divided by the pooled standard deviation, with 95% confidence intervals for d, to quantify effect sizes. Because the comparisons were exploratory, no adjustments for multiple comparisons were applied. For 2-item dimensions, inter-item correlations and Spearman-Brown coefficients are reported, as Cronbach’s alpha is less informative for 2-item scales.

## Results

### Participants

Of the 78 students, 58 (74.4%) responded to the survey. Ten were excluded from the final analysis because of incomplete responses. Thus, the final number of valid completed responses was 48. Among these respondents, 42 (87.5%) completed at least 1 activity, whereas 6 (12.5%) did not complete any.

### Main results

The results of students’ responses to the survey questionnaire are available in Dataset 1. Analysis was conducted using all valid survey responses from students who completed the brain-coloring activity and from those who did not. Internal consistency of the dimensions was evaluated using Cronbach’s alpha: perceived learning value (α=0.93), behavioral engagement (α=0.92), cognitive engagement (α=0.92), emotional engagement (α=0.89), agentic engagement (α=0.84), usability (α=0.74), and learning strategies (α=0.78). For 2-item dimensions, Spearman-Brown coefficients ranged from 0.85 to 0.92, and inter-item correlations ranged from 0.73 to 0.86, indicating coherent item pairings (Table 1).

Mann-Whitney U tests indicated that students who completed the brain-coloring activity reported significantly higher scores across all dimensions than those who did not (all P<0.05). Effect sizes (Cohen’s d) ranged from 0.65 to 1.59 across all dimensions, indicating moderate to large differences, although these findings should be interpreted cautiously given the small and unequal group sizes.

Students reported high perceived learning value when using the model. They indicated that the model improved their understanding of anatomical structures, enhanced visualization of spatial relationships, facilitated connections between lecture content, and improved their ability to recall neuroanatomy-related information.

Across the 4 engagement domains, students reported positive responses to the brain-coloring activity. Behavioral engagement reflected active exploration of the model and sustained focus during the activity. The cognitive engagement findings suggest that the model encouraged deeper thinking, reasoning, and comparison of neuroanatomical structures. The emotional engagement results indicated that students enjoyed the activity and felt motivated to learn neuroanatomy. Agentic engagement responses showed that students used the model beyond the instructions provided to enhance their learning.

Students reported that the 3D-printed model had high usability, including ease of use, an appropriate size for learning, and comprehensibility without excessive guidance. Overall, students found the model useful for learning neuroanatomy. In terms of learning strategies, the coloring activities also supported visual organization of information, and students used mental or verbal explanations to make sense of what they learned. Many students also reported that they would use the model again for future examination preparation.

## Discussion

### Key results

This study explored students’ perceptions of the learning value, usability, and learning strategies associated with a 3D-printed brain model, as well as their engagement during the neuroanatomy coloring activity. The findings suggest that students perceived high learning value and demonstrated multidimensional engagement across behavioral, cognitive, emotional, and agentic domains. Students also reported high usability of the 3D-printed model and improved learning strategies when using the model for coloring, suggesting that hands-on coloring of the 3D model may support their understanding of neuroanatomy.

### Interpretation

Students who completed at least 1 coloring activity reported greater perceived learning value and engagement than those who did not. This suggests that the coloring activity was associated with self-reported engagement, possibly reflecting the opportunity to actively integrate complex and abstract spatial information. These findings may also be understood through cognitive load theory, as actively manipulating and coloring the 3D model could support spatial understanding by helping students organize complex anatomical information more effectively. From an engagement perspective, behavioral engagement involves opportunities for students to interact with the model [10], whereas agentic engagement occurs when learners perceive that they can take initiative and influence their learning. Students who manipulated and colored the model also reported deeper cognitive processing and greater agentic involvement in the learning task.

On the other hand, students who did not complete the activities reported lower levels of behavioral and agentic engagement. This may reflect barriers to meaningful participation rather than a lack of interest in the content. Among the reasons cited for non-completion were lack of time, fear of making mistakes while coloring, and the perception that the activity was too tedious. This aligns with self-determination theory [13], which posits that engagement is influenced by learning activities that support autonomy, competence, and relatedness. For example, time constraints may diminish students’ sense of autonomy, fear of making mistakes may affect their perceptions of competence, and perceiving the task as tedious may weaken their connection to the learning activity. When these needs are insufficiently supported, students may experience reduced motivation to complete the activity.

Therefore, educators need to reduce such structural barriers to participation to promote learning. This may include allocating additional protected classroom time for the activity, providing clear step-by-step guidance, and reinforcing the formative and exploratory nature of the coloring task. In addition, the task could be designed as a collaborative activity, for example, by allowing students to work in pairs. This approach may reduce anxiety associated with making mistakes, enhance perceived competence, and foster greater engagement.

Students reported high usability of the current full-sized 3D-printed model, which was developed in response to feedback on an earlier half-sized version [2]. The larger scale enabled more precise identification of neuroanatomical structures and easier physical manipulation during coloring activities. The modular design provides instructors with flexibility to create a range of learning activities and enables students to manipulate and explore different structures from multiple perspectives. It should be noted that the earlier version was produced using stereolithography (SLA) printing, which offered higher resolution but at greater cost. Despite its lower resolution, the current FDM-printed model remained suitable for learning purposes, suggesting that lower-cost printing approaches may be sufficient for effective anatomy learning.

### Comparison with previous studies

The results of this study support existing research on 3D printing by confirming its positive impact on learning outcomes [8] and student satisfaction. A recent systematic review highlighted that such models are particularly effective for enhancing spatial understanding, recognition of structures, and student engagement in anatomy lessons [14]. Building on this literature, our findings suggest that the educational value of 3D-printed models may be further enhanced by embedding them within intentional learning activities rather than using them as standalone tools. Our study found that the coloring activity encouraged active learning, with students remaining engaged across sessions. Although we did not measure changes in academic performance, Narayanan et al. [15] found that students who participated in a neuroanatomy coloring activity scored higher than those who did not. In addition, the use of affordable FDM printing did not appear to diminish the models’ educational value, indicating that pedagogical effectiveness is not necessarily linked to higher printing costs.

### Limitations

The findings of this study are based on self-reported perceptions from a single group at a single institution and may not be generalizable to other contexts. No objective assessments of learning outcomes were conducted; therefore, the findings are limited to students’ perceptions of learning and engagement. In addition, the number of students who did not complete the coloring activity was small, resulting in a substantially imbalanced comparison. This imbalance limits the statistical stability of group comparisons, increases the risk of Type II error, and warrants caution in interpreting effect size estimates. Future research could examine the long-term impact of incorporating 3D-printed models into the curriculum and compare self-reported data with objective measures of learning. Because only 75% of the class responded to the survey, non-responders may have had different experiences or levels of engagement, potentially contributing to response bias.

### Generalizability

Coloring activities using 3D-printed models may enhance existing educational approaches for teaching complex anatomical structures. Although this study was conducted in a single cohort, the core pedagogical principles of active manipulation and spatial visualization are relevant to the study of other complex anatomical structures. An important economic insight from this research is that, despite the use of low-cost FDM printing at lower resolution than SLA, the model remained pedagogically effective when paired with well-designed learning activities.

### Suggestions

Students who completed the learning activity reported that it was beneficial to their learning. These findings suggest that educators considering the use of 3D-printed models should minimize structural barriers to engagement by providing clear instructions and sufficient classroom time to work with the models. Moreover, our findings suggest that educational value is influenced not only by the models themselves but also by the associated learning activities. Therefore, structured learning activities such as coloring may offer a practical and affordable approach to engaging learners in the study of complex anatomical structures. To address time constraints, educators should consider integrating the learning activity into the curriculum rather than treating it as an optional task, thereby improving participation and engagement.

### Conclusion

This study found that coloring a 3D-printed brain model for neuroanatomy learning was associated with positive perceptions of learning, engagement, usability, and learning strategies. Students who completed the activity reported higher engagement across behavioral, cognitive, emotional, and agentic dimensions than those who did not. This is consistent with the idea that active manipulation and intentional engagement in educational activities may support deeper involvement in the learning process. The findings suggest that coloring 3D-printed models is a practical and accessible approach that encourages active engagement with spatial information and can be readily implemented in the classroom.

From an economic perspective, the study found that a well-designed, low-cost 3D model can provide substantial learning value compared with higher-end models, particularly when it is intentionally integrated into the curriculum. Educators wishing to incorporate 3D-printed models into anatomy teaching should focus on reducing structural barriers to participation, such as time constraints and task uncertainty, by ensuring that learning activities are aligned with learning outcomes.

## Figures and Tables

**Figure f2-jeehp-23-05:**
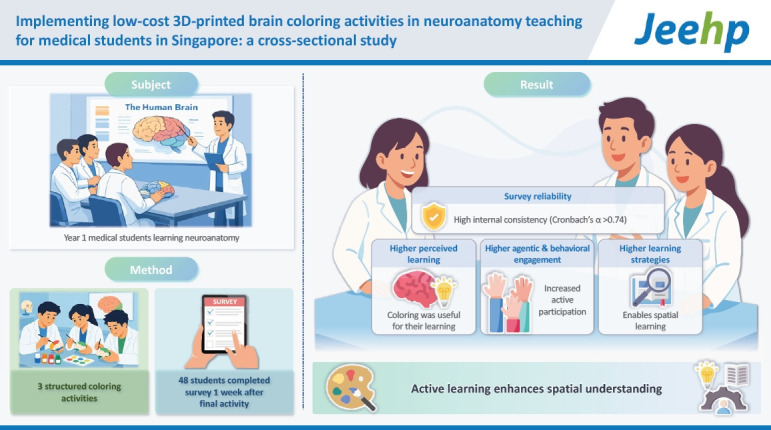


**Table 1.** Descriptive statistics and group comparison by activity completion status

Dimension	Items	α	Median		U	P-value	d	95% CI
			Completed (n=42)	Not completed (n=6)				
Perceived learning	4	0.93	4.38	3.38	50.0	0.016	0.68	−0.19 to 1.55
Behavioral engagement	2	0.92^a)^	4.00	2.50	23.5	<0.001	1.59	0.67 to 2.50
Cognitive engagement	2	0.92^a)^	4.00	3.50	55.0	0.025	0.65	−0.22 to 1.51
Emotional engagement	2	0.89^a)^	4.00	3.25	41.0	0.006	1.11	0.22 to 1.99
Agentic engagement	2	0.84^a)^	3.75	2.25	46.5	0.010	1.09	0.20 to 1.97
Usability	4	0.74	4.50	3.75	46.5	0.010	1.07	0.19 to 1.95
Learning strategies	4	0.78	4.13	3.50	49.5	0.014	1.32	0.42 to 2.21

CI, confidence interval.

a) For 2-item dimensions, Spearman-Brown coefficients are reported. Inter-item correlations: behavioral r=0.86, cognitive r=0.86, emotional r=0.82, and agentic r=0.73.

## Footnotes

**Data availability**
Data files are available from https://doi.org/10.7910/DVN/GV4U8W Dataset 1. Survey results from medical students, NUS Medical School, National University of Singapore, consisting of 20 Likert-scale items scored on a 5-point Likert scale (1=strongly disagree, 5=strongly agree) in December 2025.

## Supplementary materials

Supplement 1. Activity instructions and sample student work.

Supplement 2. Survey questions and dimensions.

Supplement 3. Audio recording of the abstract.
