# Supplementary material for: Implementing low-cost 3D-printed brain coloring activities in neuroanatomy teaching for medical students in Singapore: a cross-sectional study
Source: J Educ Eval Health Prof. 2026 Mar 18;23:5. doi: 10.3352/jeehp.2026.23.5 (PMC13054190; doi:10.3352/jeehp.2026.23.5)
Supplement: Supplementary file 2 — Supplement 1. Activity instructions and sample student work. [file jeehp-23-05-suppl1.docx]

**Supplement 1 – Activity Instruction and student work**

**Activity 1**

Using ONLY the RIGHT half of the brain:-

1. Use a dark-coloured Sharpie and mark the central sulcus
2. Use light-coloured Sharpies, colour the vascular territories

**Reflection 1**

1. How has this activity helped you in learning neuroanatomy linking structure and function?
2. Post a picture of your colouring on your MS Teams Channel together with this reflection

**Student Reflection**

**Student 1-1:** Holding and manipulating a 3D printed brain model transformed my understanding of neuroanatomy by allowing me to appreciate the true spatial relationships between structures. For example, the brainstem's orientation relative to the cerebrum and cerebellum makes more sense when viewing from multiple angles. In addition, tracing sulci and gyri with pen and physically rotating the model to see structures like the cingulate gyrus created a mental map that flat images never achieved.

**
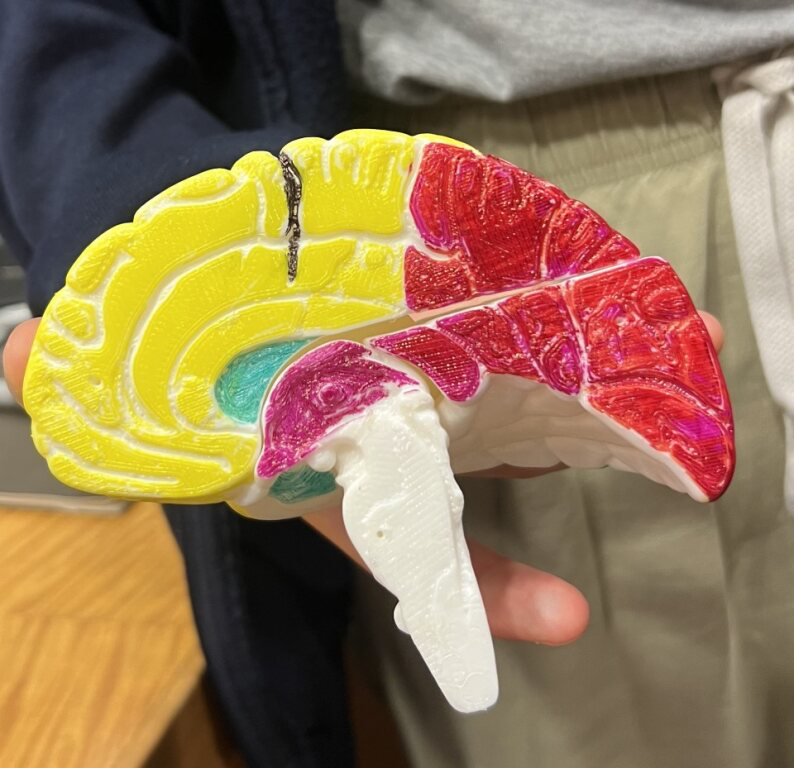
**

Figure 1: Completed Activity 1 posted on MS Teams by Student 1-1

**Activity 2**

Using ONLY the RIGHT half of the brain:-

1. Colour all the cranial nerves you can see on the model in the same colour.
2. Use different colors to color the midbrain / pons and medualla

**Reflection 2**

1. How has this activity helped you in learning neuroanatomy linking structure and function?
2. Post a picture of your colouring on your MS Teams Channel together with this reflection

**Student Reflection**

**Student 2-1**: I could see and follow the path of CN IV all the way. I also clarified the borders of the mid brain, pons, and medulla as well as the cranial nerve exits in relation to each other.


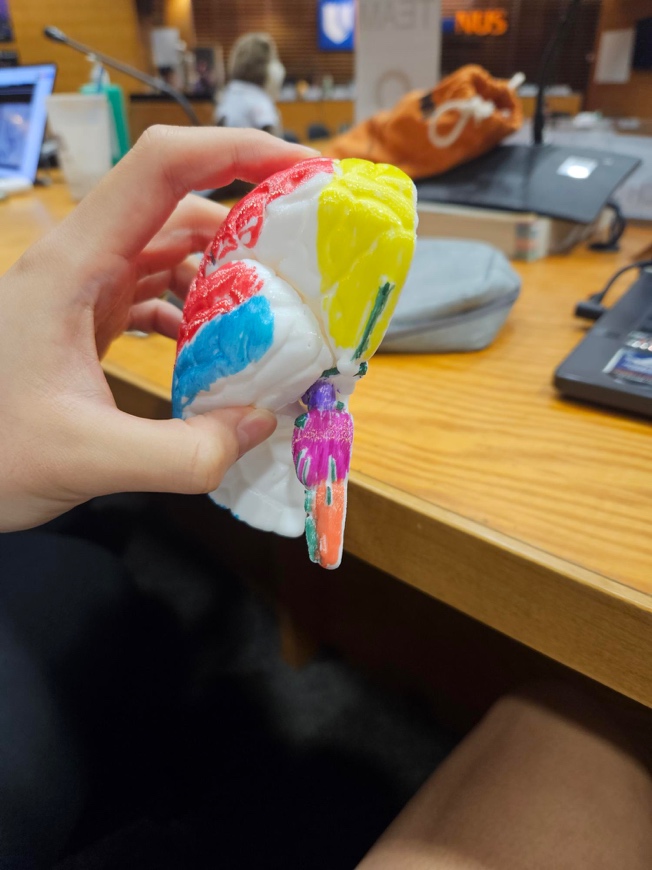


Figure 2: Completed Activity 2 posted on MS Teams by Student 2-1

**Activity 3**

Colour the following areas (either on the right or left cerebral hemisphere)

1. ⁠Primary motor cortex
2. ⁠Primary somatosensory cortex
3. ⁠Primary visual cortex
4. ⁠Brocas and Wernickes areas

**Reflection 3**

1. How has this activity helped you in learning neuroanatomy linking structure and function?
2. Post a picture of your colouring on your MS Teams Channel together with this reflection

**Student Reflection**

**Student 11-1**: It was a good revision on the functions of different parts of the cerebrum. It was especially helpful to orientate myself to the different parts of the cerebrum on the curved surface of the 3D model which is different from the 2D representations we usually see.


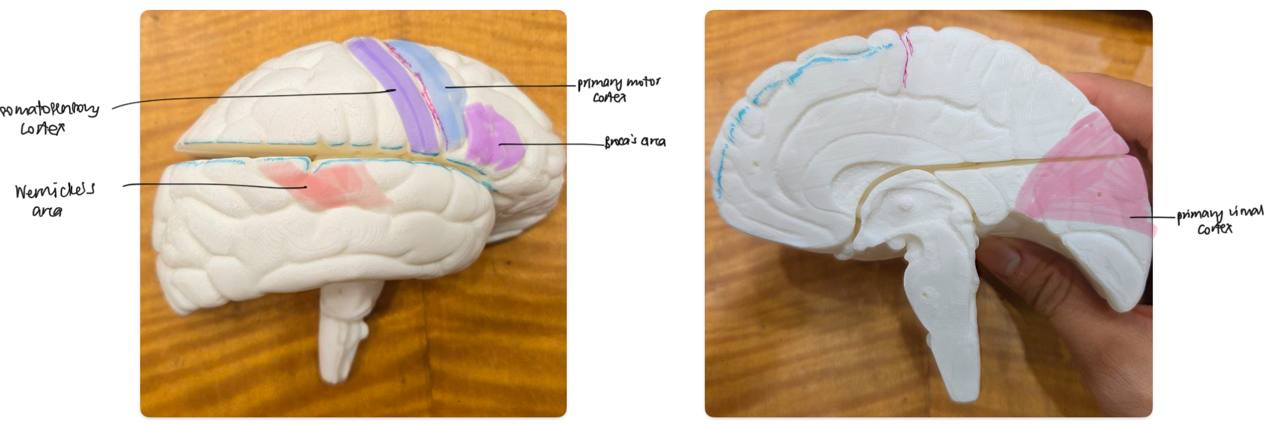


Figure 3: Completed Activity 3 posted on MS Teams by Student 11-1
