# Supplementary material for: Implementing low-cost 3D-printed brain coloring activities in neuroanatomy teaching for medical students in Singapore: a cross-sectional study
Source: J Educ Eval Health Prof. 2026 Mar 18;23:5. doi: 10.3352/jeehp.2026.23.5 (PMC13054190; doi:10.3352/jeehp.2026.23.5)
Supplement: Supplementary file 3 — Supplement 2. Survey questions and dimensions. [file jeehp-23-05-suppl2.docx]

**Supplement 2**

**Survey Question Dimensions**

**Perceived Learning Value**

1. The 3D-printed brain model helped me understand anatomical structures more clearly
2. The model helped me visualise the spatial relationships between different brain regions
3. Using the model helped me connect lecture/content knowledge to real anatomical structure
4. The model improved my ability to recall neuroanatomy information

**Behavioural Engagement**

1. I actively explored the model when colouring the model
2. The hands-on activity kept me focused on the task at hand

**Cognitive Engagement**

1. Using the model made me think more deeply about neuroanatomy
2. I found myself reasoning, comparing, or analysing as I interacted with the model.

**Emotional Engagement**

1. The model made the lesson more interesting or enjoyable
2. I felt motivated to learn more about neuroanatomy after using the model

**Agentic Engagement**

1. I used the model to help me ask questions during class
2. I took the initiative to explore the model beyond the instructions given to deepen my understanding

**Usability**

1. The model was easy to handle and manipulate (*ease of use*)
2. The model was an appropriate size and detail level for learning (*size/details*)
3. I found the model easy to understand without excessive guidance (*clarity without guidan*ce)
4. Overall, I found the model useful for learning neuroanatomy (*overall usefulness*)

**Learning Strategies**

1. The model helped me identify what I did or did not understand about neuroanatomy *(metacognitive awareness)*
2. The activity encouraged me to organise information visually (*visual organization)*
3. I used mental or verbal explanations to make sense of what I observed on the model (*self-explanation)*
4. I would use this type of model again to revise or prepare for assessments (*future strategic use*)
